# Association of activated partial thromboplastin time and fibrinogen level in patients with type II diabetes mellitus

**DOI:** 10.1186/1756-0500-6-485

**Published:** 2013-11-25

**Authors:** Binaya Sapkota, Saroj Kumar Shrestha, Sunil Poudel

**Affiliations:** 1 Kathmandu University School of Science, Dhulikhel, Kavre, Nepal; 2 Government of Nepal Civil Service Hospital, Minbhawan, Kathmandu, Nepal

**Keywords:** Activated partial thromboplastin time, Diabetes mellitus, Fibrinogen

## Abstract

**Background:**

Patients with diabetes mellitus have a high risk of atherothrombotic events. Diabetes contributes for initiation and progression of microvascular and macrovascular complications. Shortened activated partial thromboplastin time (aPTT) values may reflect hypercoaguable state, which is associated with increased thrombotic risk and adverse cardiovascular events. Increased level of fibrinogen is common in type II diabetes. The present study was conducted to study the aPTT and fibrinogen levels in diabetics in a tertiary care Teaching Hospital of Nepal.

**Methods:**

Observational study was performed at out-patients visiting Pathology Department at Tribhuvan University Teaching Hospital from August 5 to September 7, 2012. Research protocol was approved by Institutional Review Board at Tribhuvan University Institute of Medicine. Altogether 90 people who came to the hospital during study period and who met inclusion criteria were selected, out of which 72 were diabetics and 18 were normal controls. Diabetic cases were identified via verbal interview with patients themselves and review of laboratory findings and diagnosis performed by their physicians. Diabetics with a diabetic history of more than one year and stabilized with antidiabetic medicines such as insulin, metformin, glibenclamide, and gliclazide and diabetics with controlled diabetes as revealed by HbA_1c_ in the range 6.2-7% were taken for the study purpose. Data were analyzed with chi square test and Fischer’s exact test (when each cell frequency was less than 5) using Statistical Package for Social Sciences 17.

**Results:**

Maximum (53; 73.6%) diabetics and all non-diabetics had aPTT in the range 26–40 seconds. Maximum (51; 70.8%) patients had fibrinogen beyond 351 whereas all non-diabetics had fibrinogen in the range 151–350. Mean aPTT values of the diabetic patients and non-diabetic persons were 29.88 ± 4.89 seconds and 32.44 ± 2.25 seconds respectively. Mean fibrinogen values of the diabetic patients and non-diabetic persons were 388.57 ± 60.90 mg/dL and 320.89 ± 10.20 mg/dL respectively. Test data identified in results were statistically significant for aPTT (p value 0.000) and fibrinogen (p value 0.000) between the diabetics and non-diabetics.

**Conclusions:**

Diabetics have an increased level of fibrinogen and relatively shortened aPTT as compared to the non-diabetic patients.

## Background

Patients with diabetes mellitus have a high risk of atherothrombotic events. Many studies have shown a variety of diabetes mellitus related abnormalities in hemostasis and thrombosis [1,2]. The diabetic condition contributes for initiation and progression of microvascular and macrovascular complications [3].

Although modern coagulation diagnostic tests are becoming more sophisticated, standard coagulation screening tests, such as activated partial thromboplastin time (aPTT) and prothrombin time (PT) are still important basic examinations in clinical laboratories. Shortened aPTT values may reflect a hypercoaguable state, which is potentially associated with increased thrombotic risk and adverse cardiovascular events [4,5]. Shortened aPTT may result from an accumulation of circulating activated coagulation factors in plasma caused by enhanced coagulation activation in vivo [4,6]. Therefore, aPTT can be used to assess the risk of thromboembolic complications in patients with diabetes mellitus [4,7].

Plasma fibrinogen levels influence thrombogenesis, blood rheology, blood viscosity and platelet aggregation. Epidemiological studies have found a significant association between fibrinogen levels and insulin levels [8,9]. Markers of fibrinolysis are abnormal in people with metabolic syndrome and fibrinolytic dysfunction is markedly increased in subjects with diabetes mellitus and abdominal obesity [8,10]. In addition, chronic hyperglycemia and tissue glycation have marked effects on fibrin structure, clot generation and resistance to fibrinolysis [8]. Increased level of fibrinogen is common in non-insulin dependent diabetes mellitus (NIDDM) patients. In diabetic patients there is an increased rate of fibrinogen clearance, with shorter fibrinogen circulating half life [11]. Since free radicals activate thrombin formation in diabetics, oxidative stress may represent a possible link between the diabetic state and hyperfibrinogenaemia [12]. This suggests that the high level of fibrinogen in plasma will consequently shorten aPTT and might be a risk marker for cardiovascular disease because it reflects increased thrombin formation and therefore a greater possibility that a thrombotic event will occur.

Although aPTT is assayed for identifying abnormalities in the factor XII, prekallikrein and high molecular weight kininogen, intrinsic factors XI, VIII, IX and common factors X, V, prothrombin and fibrinogen pathways of coagulation, the present study has been conducted to study the APTT and fibrinogen levels in patients with type II diabetes mellitus.

## Methods

### Study site and study population

The study was performed at outpatients visiting Tribhuvan University Teaching Hospital (TUTH) Pathology Department. This was one of the renowned tertiary care teaching hospitals in Nepal that serves around 575000 outpatients annually. The target population was the diabetics with a diabetic history of more than one year. The diabetic cases were identified via verbal interview with the patients themselves and the review of the laboratory findings and diagnosis performed by their physicians. The control of blood glucose level was determined by the HbA_1c_ value 6.2-7%. Besides these, normal control samples were also collected from the patients who did not have any evidence of diabetes. Altogether 90 people who came to the hospital during the study period and who met the criteria selected for the study were chosen for the study, out of which 72 were diabetics and 18 were normal controls.

### Ethical considerations

The research protocol was approved by the Institutional Review Board at Tribhuvan University Institute of Medicine. The purpose and objectives of the study was explained to the respondents. The study protocol conformed to the ethical guidelines of the 1975 Declaration of Helsinki. Verbal informed consent from each respondent was taken. Nobody was forced to participate in the study. Confidentiality was maintained throughout research work. The respondent was allowed to quit if he/she did not want to participate in the research.

### Equipments and materials

Plastic tube with 3.2% trisodium citrate for the collection of 2 mL blood sample; centrifuge machine; 37°C water bath; micropipettes 50 microliter and 100 microliter and disposable tips; glass reaction tubes; automated blood coagulation analyzer (Sysmex CA–50®); Hemostat APTT el (human)® and Calcium chloride (CaCl_2_ 0.025 mol/l); Hemostat fibrinogen (human)®.

### Study design and study procedure

Single centric, observational study was performed at outpatients visiting Pathology Department at Tribhuvan University Teaching Hospital from August 5 to September 7, 2012. The blood sample collection of Pathology Department was chosen for the sample collection. At the time of collection, a brief interview of the patient was taken to find out their diabetic history, their blood pressure and any medicine they were taking. Patients were selected for the interview via simple random sampling technique. The purpose of the study was clearly explained to the patients. Venous blood sample was collected in 3.2% trisodium citrate vial and aPTT and fibrinogen tests were measured. The normal range for aPTT and fibrinogen were 26–40 seconds and 151–350 mg/dL respectively in the laboratory where the study was carried out. The automated blood coagulation analyzer in the laboratory exhibited the aPTT and fibrinogen values up to two decimal points whenever required for accuracy.

### Inclusion criteria

All diabetics with a diabetic history of more than one year and stabilized with antidiabetic medicines such as insulin, metformin, glibenclamide, and gliclazide were included for the study. Normal control samples were also collected from the patients who did not have any evidence of diabetes for the comparison with tests. Only the diabetic patients with controlled diabetic status as revealed by HbA_1c_ in the range 6.2-7% were taken for the study purpose.

### Exclusion criteria

Diabetics who did not wish to provide verbal consent for the participation in the interview were excluded. Diabetic patients on warfarin or heparin or any other anticoagulation therapy such as aspirin or any other medication which might affect aPTT and fibrinogen were excluded for the study. Diabetic patients with other complications were also excluded for the study.

### Procedure for aPTT determination

First of all 50 micro litre test plasma was warmed at 37°C for 5 minutes. At the same time the aPTT reagent and CaCl_2_ were also simultaneously incubated. Then 50 micro litre aPTT reagent was added to the warmed plasma and mixed and again incubated at 37°C for 3 minutes. After that 50 micro litre pre-warmed CaCl_2_ was added. Then the analyzer read the clotting time of aPTT and displayed the result in seconds.

### Procedure for fibrinogen determination

First of all the test plasma was diluted in imidazole buffered saline to give a dilution of 1:10. Then 100 micro litre of diluted plasma was warmed at 37°C for 3 minutes. Then 50 micro litre of thrombin solution was added. After that the analyzer read the clotting time and quantified the amount of fibrinogen present in the plasma.

### Blood sample collection and principle of automated blood coagulation analyzer

With an aseptic venipuncture blood sample collection technique, 2 mL of fasting sample was collected with a sterile disposable syringe. Into the 3.2% trisodium citrate vial, 1.8 mL of the collected blood sample was delivered through the wall and mixed properly without any frothing. This makes a dilution of 1:9. After mixing gently, the platelet poor plasma was obtained by centrifuging at 1500 g for 15 minutes. The test was done within 2 hours of sample collection.

Plasma was incubated for a certain time period, reagent was added. The sample to which the reagent was added was then exposed to a wavelength of 660 nm and the turbidity of the blood during the coagulation process was detected as the change in the scattered light intensity. The time recorded after the addition of calcium chloride was recorded as aPTT.

From the change in light intensity, a coagulation curve was prepared and coagulation time was found by means of the percentage detection method. The coagulation time thus formed was compared to the fibrinogen calibration chart already set in the analyzer to obtain the amount of fibrinogen in the plasma.

### Data processing and analysis

Data were processed and analyzed using Statistical Package for Social Sciences (SPSS) version 17; chi square test and Fischer’s exact test (when each cell frequency was less than 5) were used for the analysis. A p value < 0.05 was considered significant at 95% confidence interval with two-sided design.

## Results

In the study, there were 48.6% diabetic patients in the age group 41–50 years and this followed by 34.7% patients in the age group 51–60 years. The study showed that 55.6% were non-diabetics in the age group 51–60 years. The study revealed that 62.5% diabetics were males and 55.6% non-diabetics were also males (Table 1).

**Table 1:** Basic characteristics of study population

**Variables**	**Frequency**	**Percentage**
**Age of diabetic patients (in years)**		
<=40	3	4.2
41-50	35	48.6
51-60	25	34.7
61-70	5	6.9
71-80	4	5.6
**Age of non-diabetic patients (in years)**		
<=40	1	5.6
41-50	5	27.8
51-60	10	55.6
61-70	2	11.1
**Gender of diabetic patients**		
Male	45	62.5
Female	27	37.5
**Gender of non-diabetic patients**		
Male	10	55.6
Female	8	44.4

(n = 90: case = 72; control = 18) (Mean age of diabetic patient =51.53, SD 8.71 years; mean age of non-diabetic patients =52.72, SD 7.41 years).

The study showed that 72% diabetic patients had history of diabetes of 2–6 years and 56.9% diabetic patients were taking antidiabetic medications. Among the patients, 68.1% had normal blood pressure (Table 2).

**Table 2:** Diabetes-related characteristics of the diabetic patients

**Variables**	**Frequency**	**Percentage**
**Duration of diabetes mellitus (in years)**		
2-6	64	88
7-11	5	7
12-16	2	3
17-21	1	2
**Antidiabetic medication used by diabetic patients**		
No	31	43.1
Yes	41	56.9
**Blood pressure of diabetic patients (in mm Hg)**		
Normal	49	68.1
High	23	31.9

(case =72).

**Figure 1:**
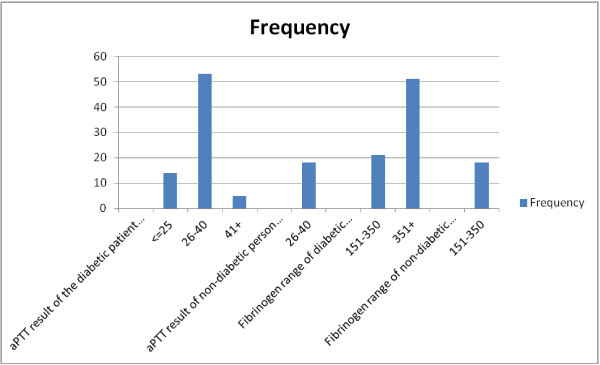
**The aPTT and Fibrinogen related characteristics of the study population.** (Mean aPTT of diabetics and non-diabetic person **=**29.88 ± 4.89 seconds and 32.44 ± 2.25 seconds respectively; p value 0.000) (Mean fibrinogen level of diabetics and non-diabetic person =388.57 ± 60.90 mg/dL and 320.89 ± 10.20 mg/dL; p value 0.000).

The study found that 73.6% diabetic patients had aPTT in the range 26–40 seconds whereas 100% of the non-diabetic persons had aPTT in that range. Maximum (70.8%) patients had fibrinogen in beyond 351 whereas 100% non-diabetic persons had fibrinogen in the range 151–350. The chi square and Fischer’s exact tests showed that the test data were statistically significant for aPTT (p value 0.000) and fibrinogen (p value 0.000) between the diabetics and non-diabetics (Figure 1).

The chi square and Fischer’s exact tests showed that there was no significant association between duration of diabetes mellitus and aPTT of diabetic patients (p value 0.206) and no significant association between duration of diabetes mellitus and fibrinogen level of diabetic patients (p value 0.221).

## Discussion

In patient with diabetes mellitus, persistent hyperglycemia exposes red blood cells to elevated glucose concentration, resulting in glycation of hemoglobin, prothrombin, fibrinogen and other protein involved in clotting mechanisms.

The present study showed that the association of aPTT with duration of the diabetes was not significant (p > 0.05). Zhao et al. observed that when diabetic group was compared with normal group, statistically significant differences were observed in overall aPTT (p =0.001) [13].

Fibrinogen levels have been shown to be elevated in type II diabetes mellitus patients [14]. Similarly, Lippi et al. also observed that there were significantly higher fibrinogen levels and shortened aPTT values in diabetic patients. The exact biological mechanisms of thrombosis in diabetics are likely to be multifactorial and incompletely understood yet [4]. Korte et al. found that patients presenting with shortened aPTT values were in a complex hypercoagulant state and at increased risk for thromboembolism [15]. Tripodi et al. found that hypercoaguability detected by shortened aPTT values was independently associated with venous thromboembolism and hypothesized that shortened aPTT could be as a risk matter for venous thromboembolism (VTE) [7]. Shortened aPTTs are generally considered to be laboratory artifacts arising from problematic venipunctures [16]. Shortened aPTT may result from an accumulation of circulating activated coagulation factors in plasma caused by enhanced coagulation activation in vivo [4,6].

### Study limitation

The study was limited to a single hospital only. The sample size might not be the exact representatives of the whole case so as to generalize the findings of the study. The low ratio of non-diabetic to diabetic patients was due to the fact that less number of study population came to the study site during the study period.

## Conclusions

The patients suffering from type II diabetes mellitus were found to have an increased level of fibrinogen and relatively shortened aPTT as compared to the non-diabetic patients.

## Competing interests

The authors declare that they have no competing interests.

## Authors’ contributions

BS designed the study, performed literature review, analyzed and interpreted the data, and prepared the final manuscript. SKS designed the study, performed literature review, collected data and drafted the manuscript. SP contributed to perform literature review, collect data and draft the manuscript. All authors read and approved the final manuscript.
